# Evaluation of a Standardized Extract Obtained from Cashew Apple (*Anacardium occidentale* L.) Bagasse in DSS-Induced Mouse Colitis

**DOI:** 10.3390/foods12173318

**Published:** 2023-09-04

**Authors:** Gisele Goulart da Silva, Lucia Elaine de Oliveira Braga, Ellen Cristina Souza de Oliveira, João Ernesto de Carvalho, Josy Goldoni Lazarini, Pedro Luiz Rosalen, Ana Paula Dionísio, Ana Lucia Tasca Gois Ruiz

**Affiliations:** 1 Piracicaba Dental School, Graduate Program in Dentistry, University of Campinas, UNICAMP, Piracicaba 13414-903, SP, Brazil; giselegoulart87@gmail.com (G.G.d.S.); naine17@hotmail.com (L.E.d.O.B.); rosalen@fop.unicamp.br (P.L.R.); 2 Institute of Biology, Cellular and Structural Biology Graduate Program, University of Campinas, UNICAMP, Campinas 13083-865, SP, Brazil; ellen_oliveira87@yahoo.com.br; 3 Faculty of Pharmaceutical Sciences, University of Campinas, UNICAMP, Campinas 13083-871, SP, Brazil; carvalho@fcf.unicamp.br; 4 Department of Physiological Sciences, Piracicaba Dental School, University of Campinas, UNICAMP, Piracicaba 13414-903, SP, Brazil; josy662@hotmail.com; 5 Faculty of Medicine, Universidade Anhembi Morumbi, Piracicaba 13425-380, SP, Brazil; 6 Biological Sciences Graduate Program, Federal University of Alfenas, UNIFAL-MG, Alfenas 37130-001, MG, Brazil; 7 Embrapa Agroindústria Tropical, Fortaleza 60511-110, CE, Brazil; ana.dionisio@embrapa.br

**Keywords:** *Anacardium occidentale* L., food by-products, carotenoids, anacardic acids, colitis, inflammatory bowel diseases

## Abstract

Inflammatory bowel diseases (IBD) include Crohn’s disease and ulcerative colitis. Several studies relate eating habits to different aspects of IBD, such as progression and worsening of the clinical condition. Therefore, many natural products (NPs) such as polyphenols and carotenoids have been identified as promising agents in supporting IBD. An interesting source for obtaining bioactive NPs is the by-products of the food industry. The present study evaluated the potential beneficial effect of a standardized extract (CAE) obtained from cashew apple bagasse in the dextran sulfate sodium (DSS)-induced ulcerative colitis model in mice. This was the first time that CAE had been evaluated in this experimental model. Chemical evaluation of CAE identified carotenoids (96.28 ± 0.15 mg/100 g), phenolic compounds (37.49 ± 0.64 mg/100 g), and a mixture of anacardic acids (C15:3 = 94.2 ± 0.6 mg/100 g; C15:2 = 108.4 ± 0.1 mg/100 g; C15:1 = 214.8 ± 0.2 mg/100 g). Administration of CAE (500 mg/kg, 4 days, p.o.) after DSS challenge was more effective in delaying disease progression compared with prior treatment (500 mg/kg, 30 days, p.o.), according to the disease activity index. However, no treatment strategy with CAE was able to prevent or inhibit disease progression, since all parameters evaluated (macroscopic, biochemical, and histopathological) in CAE-treated animals were similar to those observed in DSS-challenged animals. Despite the high dose (500 mg/kg), the standardized extract (CAE) did not result in an effective concentration of carotenoids. Furthermore, as some anacardic acids have been reported as histone acetyltransferases inhibitors, there could be a possible antagonistic relationship between carotenoids and anacardic acids. Complementary research will be necessary to test the hypothesis of antagonism. Thus, an optimized extract, with an even higher concentration of carotenoids, obtained from cashew apple bagasse, can be developed as a possible adjuvant food supplement for inflammatory bowel diseases.

## 1. Introduction

Characterized by chronic and recurrent inflammation of the intestinal wall, inflammatory bowel diseases (IBD) comprise Crohn’s disease and ulcerative colitis [1]. Several factors are described as contributing to the IBD development, such as genetics and environmental factors. Among the environmental factors, eating habits seem to be strongly associated with the development and progression of IBD [2,3,4].

Currently, no effective cure for IBD is available. The therapeutic strategy is based on relieving acute symptoms, promoting disease remission, and preventing recurrences. First-line drugs include aminosalicylates, corticosteroids, immunosuppressant, and antibiotics. The most severe cases can be treated with so-called biological therapy, usually anti-TNF-α antibodies [5,6].

Different studies have demonstrated the beneficial relationship between inflammatory process resolution and the consumption of fruits and vegetables, both due to the presence of secondary metabolites and the nutritional constituents [6,7,8,9]. Widely found in nature, carotenoids provide colors ranging from yellow to red to fruits, vegetables, and animals. All carotenoids found in human plasma come from food [10]. Chemically classified as carotenes and xanthophylls, provitamin A carotenoids are those that can be metabolized in vivo into vitamin A, such as β-carotene and β-cryptoxanthin [10,11]. Some clinical trials have already demonstrated the beneficial effects of carotenoid consumption by patients with IBD [12,13]. Derived from primary and secondary activities of agro-industrial processes [14], by-products of different plants, such as cashew, constitute an interesting source of carotenoids [15,16,17].

Popularly known as the cashew tree, *Anacardium occidentale* L. (Anacardiaceae family) is a tropical tree native to South America and cultivated in several tropical areas of the world [18,19,20]. In addition to traditional medicinal uses for the treatment of wounds, ulcers, dyspepsia, diarrhea, and diabetes [20], cashew nuts (true fruit), apple (pseudo fruit), gum, and wood represent an important source of income [18,19]. An environmental concern in the production of cashew juice is the large amount of bagasse (about 300 thousand tons of bagasse per year in Brazil) generated in the process. These fibers are popularly used as animal feed or simply discarded. Several studies have demonstrated the potential of cashew bagasse as a source of renewable energy and bioactive substances [17,21,22]. In this context, Brazilian researchers from Embrapa (CE, Brazil) in partnership with CIRAD (France) developed a method based on green techniques (such as pressing and filtration) to prepare an aqueous extract (CAE) with an intense yellowish color from cashew apple bagasse [23,24]. Chemical analysis of CAE showed the presence of carotenoids, anacardic acids, and phenolic compounds (Table 1) [23,24,25,26].

After checking the no-observed-adverse-effect level (NOAEL) in different preclinical models and the potential use as food dye and as a gastro-protective agent [25,26,27,28], the present study evaluated whether CAE could have some beneficial effects in the acute ulcerative colitis, using the dextran sulfate sodium-induced colitis model in mice.

## 2. Materials and Methods

### 2.1. Plant Material

The aqueous extract (CAE) was prepared from the cashew apple (*A. occidentale*, variety CCP-76) bagasse in water (1:1 *w*/*w*) using environmentally friendly techniques (pressing and filtration) followed by lyophilization, with a yield of 3 to 4%, as previously described [23,24,26]. The chemical characterization of CAE using HPLC-DAD-MS, UPLC-QToF-ESI, and colorimetric methods (Table 1) have been previously reported [24,25,26]. Packaged in amber glass vials, CAE was kept at −80 °C before pharmacological evaluation. The project was registered at the National System for the Management of Genetic Heritage and Associated Traditional Knowledge (SisGen, # A1CBB10).

### 2.2. Reagents

The following reagents were obtained: ketamine (Dopalen injectable^®^, Paulínia, Brazil), xylazine (Anasedan injectable^®^, Paulínia, Brazil), Tween 80 (Synth^®^, Diadema, SP, Brazil), hexadecyltrimethylammonium bromide (HTAB) (Sigma Aldrich^®^ H5882, Steinheim, Germany), o-dianisidine dihydrochloride (Sigma Aldrich^®^ D3252, Steinheim, Germany), dextran sulphate sodium salt (DSS)—colitis grade (36,000–50,000 MW, cod. 02160110-CF, MP Biomedicals, LLC, Solon, OH, USA), hydrogen peroxide (Synth^®^), horseradish peroxidase (Sigma Aldrich^®^ P8250, Steinheim, Germany), trichloroacetic acid (TCA) (Sigma Aldrich^®^ T4885, Steinheim, Germany), reduced L-glutathione (Sigma Aldrich^®^ G-4251, Steinheim, Germany), β-nicotinamide adenine dinucleotide 2′-phosphate reduced tetrasodium salt hydrate (NADPH) (Sigma Aldrich^®^ N-7505, Steinheim, Germany), 5,5′-dithiobis (2-nitrobenzoic acid) (DTNB) (Sigma Aldrich^®^ D-8130, Steinheim, Germany), and buffer RIPA (Sigma Aldrich^®^ R0278, Steinheim, Germany). IL-1β mouse, IL-10 mouse, CXCL-MIP-2 mouse, and TNF-α mouse were obtained from R&D Systems^®^, Inc. (Minneapolis, MN, USA).

### 2.3. In Vivo Pharmacological Evaluation

#### 2.3.1. Animals 

Specific pathogen-free C57BL/6J female mice (4 weeks old, forty animals) were obtained from the Multidisciplinary Center for Biological Research in the Area of Science in Laboratory Animals at UNICAMP (CEMIB/UNICAMP). The project was approved by the UNICAMP Committee for Ethics in the Use of Animals (CEUA/UNICAMP), protocol number 4725-1/2017. Animal care, as well as protocols, were carried out in accordance with the principles and guidelines adopted by the Brazilian National Council for Animal Experiment Control (CONCEA) and the European Union Regarding Animal Experimentation (Directive of the European Counsel 86/609/EC). The animals (5 animals/cage) remained throughout the period in polyethylene boxes (matte white), at a temperature of 22 ± 2 °C, a relative humidity of 50 ± 20%, and a light/dark cycle of 12/12 h. All animals received Nuvilab^®^ commercial pelleted feed and drinking water ad libitum. Food consumption was not monitored during the experiment. Water consumption was monitored per cage every day during the DSS challenge (30th to 37th experimental day, Table S1 in Supplementary Material). At the beginning of the experiment, all animals were 8 weeks old (body weight 18–22 g). Euthanasia was performed by deep anesthesia (intraperitoneal route, ketamine:xylazine 300 mg/kg:30 mg/kg) followed by cervical dislocation.

#### 2.3.2. Sample Preparation

CAE was mixed with Tween 80 followed by dilution in PBS (0.2 M phosphate buffer solution, pH 7, in 0.9% NaCl solution), resulting in doses of 100 and 500 mg/kg body weight. The final concentration of Tween 80 was 0.2%. Oral treatment was performed by gavage (5 mL/kg) using a flexible orogastric catheter (Ø 2 mm, 5 cm length).

#### 2.3.3. Dextran Sulfate Sodium (DSS)-Induced Acute Colitis Model

**Figure 1:**
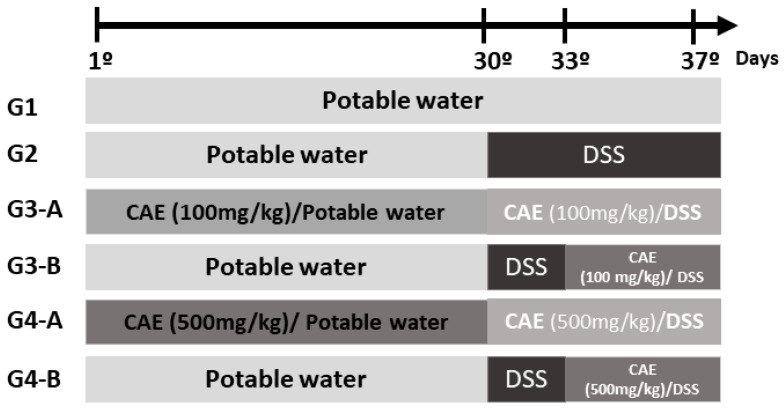
Treatment protocol during the DSS-induced acute colitis model. Animals: C57BL/6J female mice (8 weeks). Groups: G1 = 5 animals accommodated in one cage; G2–G4 = 5 animals per cage, totaling 7 cages (*n* = 7 animals/group). Treatments: G1 = drinking water ad libitum (satellite group); G2–G4 = dextran sulfate sodium group 3% (DSS, *ad libitum*, 30th to 37th experimental day); G3-A and G4-A = CAE at 100 and 500 mg/kg, respectively, from the 1st to the 37th experimental day; G3-B and G4-B = CAE at 100 and 500 mg/kg, respectively, from the 33rd to the 37th experimental day.

Following the protocol described by de Oliveira Braga et al. [29] with few adaptations, forty C57BL/6J female mice were randomly assigned to six experimental groups. Group G1 (satellite, *n* = 5 animals/group) received drinking water ad libitum without any treatment, while animals in groups G2–G4 (DSS challenge, *n* = 7 animals/group) received dextran sulfate sodium solution (DSS, 3% in drinking water *ad libitum*) from the 30th to the 37th experimental day. As shown in Figure 1, treatment with CAE was performed by gavage from the 1st to the 37th experimental day (100 and 500 mg/kg, *n* = 7 animals/group, G3-A and G4-A, respectively) or from the 33rd to the 37th experimental day (100 and 500 mg/kg, *n* = 7 animals/group, G3-B and G4-B, respectively). Housing five animals per cage, there was one animal from each treatment in each cage, resulting in seven cages for DSS-challenge groups. Considering the concern to reduce the number of animals per experiment and that there is no gold standard for the treatment of IBD, it was decided not to include a group as a positive control.

Body weight was assessed on experimental days 0 (baseline), 7, 14, 21, and 28, before the DSS challenge. During the DSS challenge (30th to 37th experimental day), the animals were clinically evaluated for body weight loss (0 = no weight loss; 1 = 1% ≤ weight loss < 5%; 2 = 5% ≤ weight loss < 10%; 3 = weight loss ≥ 10%), presence of secretion or blood in the anal area (0 = absent; 1 = clear secretion; 2 = reddish secretion; 4 = anal bleeding), and stool consistency (0 = normal stools; 2 = soft pellet stools; 3 = liquid stools). These parameters were used to calculate the disease activity index (DAI) [29,30]. 

On the 37th experimental day, all animals were euthanized for colon removal (from the ileocecal junction to the anus). After evaluating the length with the aid of a ruler, each colon was washed with PBS, photographed, and cut longitudinally into fragments for histological and biochemical analysis [myeloperoxidase (MPO) activity; reduced glutathione; TNF-α, IL-1β, IL-10, and CXCL2/MIP-2 levels] following the same protocols described by de Oliveira Braga et al. [29]. 

### 2.4. Statistical Analysis 

The results were expressed as mean ± standard error or as boxplot graphs. Statistical analysis of variance (Kruskal–Wallis followed by Dunn’s test for qualitative analyses, one-way ANOVA followed by Dunnet’s or Tukey’s tests, and two-way ANOVA followed by Bonferroni’s test for quantitative analysis) was performed to establish whether the differences observed among the groups were significant (*p* ≤ 0.05) or not, using the GraphPad Prism software version 6.0 (GraphPad Software).

## 3. Results

**Figure 2:**
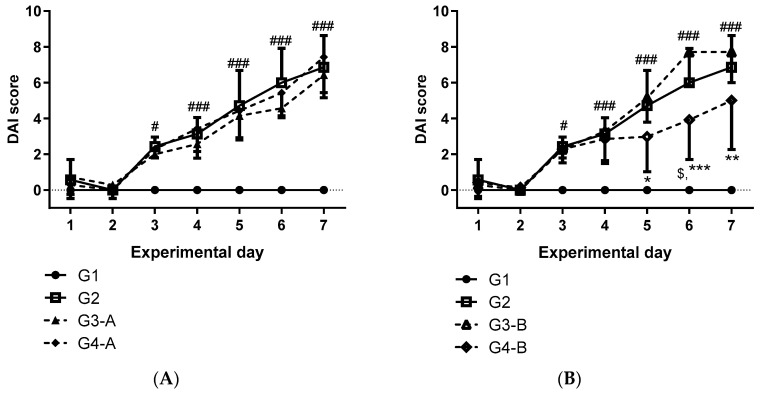
DAI evolution during DSS-induced colitis model in female C57BL/6J mice. (**A**) DAI evolution after 37-day CAE treatment at 100 (group G3-A) and 500 mg/kg (group G4-A); (**B**) DAI evolution after 4-day CAE treatment at 100 (group G3-B) and 500 mg/kg (group G4-B). Results expressed as mean ± standard deviation. Animals: female C57BL/6J mice (8 weeks). Groups: G1—satellite group; G2—DSS-challenge group; G3-A and G4-A—37-day dose-repeated CAE treatment at 100 and 500 mg/Kg, respectively, plus DSS challenge; G3-B and G4-B—4-day dose-repeated CAE treatment at 100 and 500 mg/Kg, respectively, plus DSS challenge. Statistical analysis by two-way ANOVA followed by Bonferroni’s test (# *p* < 0.05, ### *p* < 0.001 in comparison to the satellite group; $ *p* < 0.05 in comparison to DSS group; * *p* < 0.05, ** *p* < 0.01, *** *p* < 0.001 in comparison to 4-day CAE treatment at 100 mg/kg).

Considering the chemical composition of CAE (Table 1) and the average weight of the mouse as 20 g, doses of 100 and 500 mg/kg of CAE provided 1.93 and 9.63 µg/mouse/day of total carotenoids, respectively (Table 2). After 30 days of treatment with CAE, C57BL/6J mice from groups G3-A and G4-A (100 and 500 mg/kg, respectively) showed a significant reduction in body weight gain compared to untreated animals (G1, G2, G3-B, and G4-B). During the first 30 experimental days, only animals from groups G3-A and G4-A received gavage (Table 3). Regardless of dose range, the 37-day dose-repeated CAE treatment did not prevent the worsening of colitis (Figure 2A, groups G3-A and G4-A) while the 4-day dose-repeated CAE treatment, at 500 mg/kg (group G4-B), reduced (*p* < 0.05) the worsening of colitis symptoms at the sixth DSS-challenge day compared to DSS-challenge animals (group G2) (Figure 2B). However, none of the CAE treatment strategies was able to prevent the disease progression (*p* > 0.05) (Figure 2).

**Figure 3:**
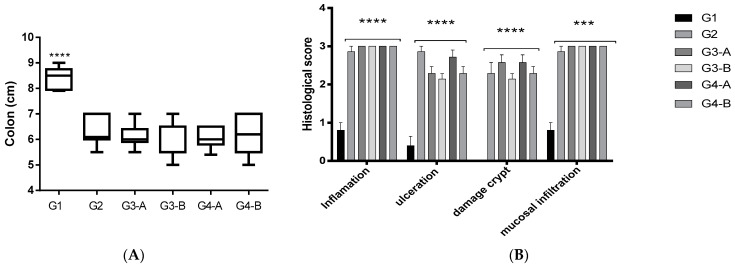
Effect of CAE on macroscopic (colon length) and microscopic (histopathological) aspects of mice colon in the DSS-induced colitis model in C57BL/6J mice. (**A**) Colon length results (from ileocecal junction to anus) expressed as mean ± standard error (statistical analysis by ANOVA One-way followed by Dunnet’s test, **** *p* < 0.0001 compared to group G2). (**B**) Histologic score for inflammation, ulceration, crypt damage, and mucosal infiltration (statistical analysis of variance by Kruskal–Wallis test followed by Dunn’s test, *** *p* < 0.001, **** *p* < 0.0001 compared to group G1). Groups: G1—satellite group; G2 = DSS-challenge group; G3-A and G4-A—37-day dose-repeated CAE treatment at 100 and 500 mg/Kg, respectively, plus DSS challenge; G3-B and G4-B—4-day dose-repeated CAE treatment at 100 and 500 mg/Kg, respectively, plus DSS challenge.

The DSS challenge led to colon shortening along with the presence of ulcerative lesions (G2, Figure 3, Figures S1 and S2 in Supplementary Material) compared to satellite group G1. Regardless of dose range and treatment scheme (groups G3-A/B and G4-A/B), CAE treatment was unable to prevent or reverse colon shortening and ulceration (Figure 3, Figures S1 and S2 in Supplementary Material).

**Figure 4:**
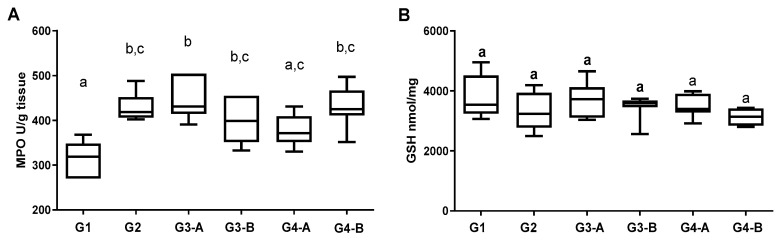

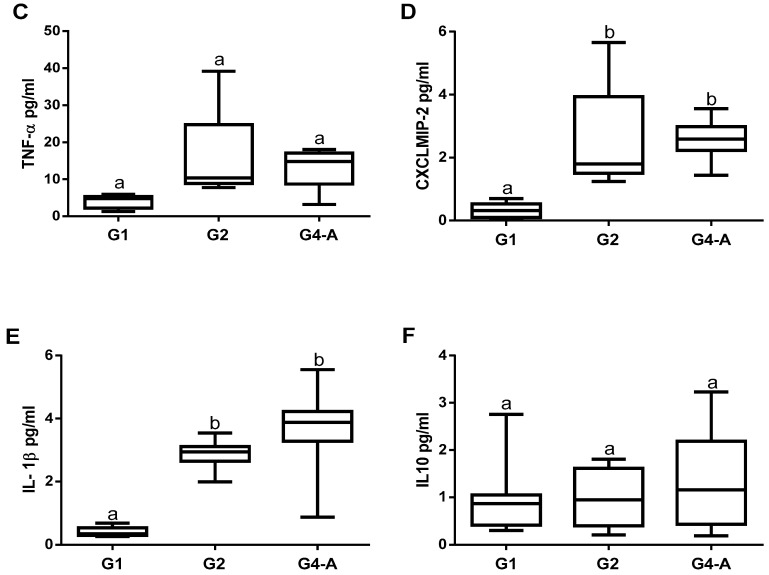
Biochemical analysis inflammatory evaluation in the mice’s colons in the DSS-induced colitis model. (**A**) Myeloperoxidase activity (MPO U/g tissue); (**B**) reduced glutathione level (GSH mmol/mg tissue); (**C**) concentration of TNF-α (pg/mL); (**D**) concentration of CXCL-MIP2 (pg/mL); (**E**) concentration of IL-1β (pg/mL); (**F**) concentration of IL-10 (pg/mL). Results expressed as a boxplot graphic. Animals: female C57BL/6J mice (8 weeks). Groups: G1—satellite group; G2—DSS-challenge group; G3-A and G4-A—37-day dose-repeated CAE treatment at 100 and 500 mg/Kg, respectively, plus DSS challenge; G3-B and G4-B—4-day dose-repeated CAE treatment at 100 and 500 mg/Kg, respectively, plus DSS challenge. Statistical analysis by ANOVA one-way followed by Tukey’s test (different letters indicate significant differences between groups, *p* ≤ 0.05).

Histopathological analysis (Figure 3B and Figure S2, in Supplementary Material) showed DSS induction of crypt damage and ulcerations, along with infiltration of inflammatory cells (group G2, Figure 3B and Figure S2, in Supplementary Material) compared to the colonic tissues of satellite animals (group G1, Figure 3B and Figure S2, in Supplementary Material). Corroborating macroscopic and biochemical findings (increased MPO activity, CXCL/MIP-2, TNF-α, and IL-1β levels, Figure 4), CAE was not able to prevent (G3-A and G4-A) or reverse (G3-B and G4-B) the DSS-induced tissue damage (Figure 3B and Figure S2, in Supplementary Material).

## 4. Discussion

The present study evaluated the potential beneficial effect of the carotenoid- and anacardic-acid-enriched aqueous extract (CAE), obtained from the cashew apple bagasse, a by-product of cashew juice production, on DSS-induced colitis in mice. As CAE modulated the gastric inflammatory process in a dose-repeated acetyl salicylic acid (ASA)-induced gastric lesion model in rats [28], it was important to assess whether these effects could be similar in intestinal inflammation. The dose range was established based on the effective dose (100 mg/kg) in a dose-repeated ASA-induced gastric lesion model in rats [28], and the maximum tolerable dose (500 mg/kg) evidenced by toxicological study in mice [27]. The treatment regimen sought to mimic the daily use of a dietary supplement before (37-day dose-repeated) and after (4-day dose-repeated) the onset of colitis symptoms.

Considering body weight evolution (Table 3), on the 30th experimental day, dose-repeated CAE treatment promoted a slightly lower weight gain in C57BL/6J mice, regardless of dose, compared to animals in the other groups. Some studies associated the consumption of cashew apple extracts or juice with changes in body weight. In preclinical studies, both consumption of ethanolic cashew apple extract (400 mg/kg, p.o., 60-day treatment) or ethanolic extract of cashew apple bagasse (Cashwin^®^, 200 mg/kg, p.o., 8-week treatment) prevented diet-induced obesity in rats and mice, respectively [31,32]. In a clinical trial, cashew juice consumption by trained and untrained men (3.5 mL/kg, 4 weeks) promoted an increase in fat expenditure during exercise [33]. These results were attributed to the total phenolic content, including tannins and anacardic acids, of cashew apple [31,32,33]. 

Despite this, the 30-day dose-repeated oral toxicity evaluation of CAE in Swiss female mice did not show any influence on the body weight evolution [27]. This evidence seems to suggest that cashew apple juice or extracts may preferentially modulate body weight in obese individuals or during exercise, without affecting normal weight individuals. As only animals from groups G3-A and G4-A were submitted to gavage for 30 days, the observed reduction in body weight gain may be related to gavage-induced stress. During handling, mice may feel threatened and some events, such as increased body temperature and metabolism, may occur, resulting in lower body weight gain [34,35].

The dextran sodium sulfate (DSS)-induced model is one of the most used protocols of chemically induced colitis. Administered with drinking water, DSS promotes an acute colitis manifestation that resembles human ulcerative colitis [36]. DSS-induced epithelial barrier disruption results in increased infiltration of innate (neutrophils and macrophages) and adaptive (B and T cells) immune cells, along with higher levels of TNF-α, IL-1β, and CXCL2/MIP-2 at colonic tissues [36,37]. Acting as antimicrobial agent, the exacerbation of MPO activity leads to increased ROS production with consequent depletion of endogenous antioxidant substances such as reduced glutathione (GSH) [38]. Clinically, colitis results in symptoms such as diarrhea, blood in the stool, and body weight loss. In the DSS-induced colitis model, these clinical parameters can be scored to provide the disease activity index (DAI) [36,39]. Along with the progressive increase in DAI (Figure 2), tissue damage was evidenced by the macroscopic (reduction in intestinal length) and histopathological findings (ulcerative regions, crypt damage, and cellular infiltration in the mucosa) observed in DSS-treated animals (Figure 3 and Figure S2 in Supplementary Material).

Among the micro-molecules with beneficial effects on IBD due to their anti-inflammatory, antioxidant, or prebiotic effects, the carotenoids stand out [2,3,7,8,10,11,12,13].

Carotenoids can regulate the inflammatory response by modulating the expression of transcription factors and the secretion of cytokines and chemokines, along with ROS scavenging effects. Furthermore, it has been postulated that carotenoids can act directly on the microbiota. These effects contribute to the maintenance of intestinal homeostasis [11,13,14]. In humans, consumption of a carotenoid-enriched diet may alleviate some symptoms of ulcerative colitis during the remission phase. Diets containing higher amounts (between 3.3 and 4.1 mg/day) of non-provitamin A carotenoids, such as lycopene, lutein, and zeaxanthin, were associated with lower incidence of blood, mucus, and pus in the stool [16]. Based on this clinical trial and the dose conversion formula [40], the effective dose of non-provitamin A carotenoids mixture in mice can be estimated as 13 µg/day/mouse.

Composed of approximately 77% of non-provitamin A carotenoids, the total carotenoid content of CAE (≈96 mg/100 g, Table 1) was almost 160x higher than that described for cashew apple pulp (0.6 mg/100 g) [24]. At 500 mg/kg, CAE intake provided 7.4 µg/day/mouse of non-provitamin A (Table 2), which may explain the slight and significant improvement observed in DAI (4-day treatment) and MPO activity (37-day treatment). This evidence suggests that, regardless of treatment time, the amount of carotenoids provided by 500 mg/kg of CAE promoted some beneficial effect. However, it was not enough to prevent or reverse the course of acute colitis induced by DSS, as observed by macroscopic (colon length reduction), biochemical (increased IL-1β, TNF-α, and CXCL2/MIP-2), and histopathological analyzes of colonic tissues performed at the end of the experiment.

As evidenced by some studies, intestinal inflammation can negatively affect absorption and metabolism of carotenoids. Along with the cell damage in the large intestine, pro-inflammatory cytokines induced by ulcerative colitis can affect enterocytes. This condition results in decreased serum levels of bioactive carotenoid derivatives [7,41,42,43]. Thus, in addition to offering a lower dose (1.48 and 7.4 µg/day/mouse for 100 and 500 mg/kg of CAE) of carotenoids than that estimated as effective (13 µg/day/mouse), the previous treatment with CAE did not result in a carotenoid content capable of preventing the deleterious effects of DSS. Furthermore, already established inflammation in the colon may have impaired carotenoid absorption when CAE was administered after starting the DSS challenge. Thus, under the experimental conditions used (two doses and two treatment regimens), it was not possible to determine the effective concentration of CAE needed to reverse the signs and symptoms of colitis by 50% (IC_50_).

In addition, a mixture of 6-[8′(Z),11′(Z),14′-pentadecatrienyl] salicylic acid (anacardic acid C15:3), 6-[8′(Z),11′(Z)-pentadecadienyl] salicylic acid (anacardic acid C15:2), and 6-[8′(Z)-pentadecenyl] salicylic acid (anacardic acid C15:1), in a relative ratio of 1:1:2, was identified in CAE [26]. Found in *Anacardium* and *Ginkgo* species, anacardic acids are long-chain phenolic acids derived from 6-alkylsalicylic acid [19,44]. One of them, 6-pentadecylsalicylic acid, is a nonspecific inhibitor of histone acetyltransferase (HAT) affecting DNA transcription and modulating the NF-κB pathway [45,46]. An epigenetic study demonstrated the downregulation of histone acetyltransferase KAT2B as a common alteration in IBD patients. By inhibiting IL-10 expression, this downregulation may disrupt the immune response at the intestinal epithelium during IBD, worsening the clinical condition [47].

Therefore, the lack of beneficial effects of CAE in DDS-induced colitis may be related to a lower-than-estimated non-provitamin A carotenoid content and impaired carotenoid absorption, together with the potential inhibition of HATs induced by anacardic acids. Studies aimed at isolating carotenoids and anacardic acids present in CAE together with the evaluation of these substances in the DSS-induced colitis model will be necessary to elucidate these hypotheses.

Finally, in DNBS-induced colitis in CD1 mice, the whole ground cashew nuts (100 mg/kg, in NaCl 0.9%, 4-day oral treatment) partially reduced macroscopic, histopathological, and biochemical parameters of colonic inflammation. These cashew nuts were characterized in terms of nutritional composition without evaluating the anacardic acid content [48]. Considering the differences in experimental model (DNBS-induced versus DSS-induced colitis), in mouse strain (CD1 mice versus C57BL/6J mice), and in chemical composition between cashew nuts and CAE, it is not possible to establish a direct comparison between the studies.

## 5. Conclusions

For the first time, it was demonstrated that the carotenoid- and anacardic-acid-enriched aqueous extract (CAE) obtained from cashew apple bagasse was able to delay the worsening of clinical signs without preventing DSS-induced acute colitis evolution. Future studies aiming at the chemical separation of anacardic acids and carotenoids from CAE will be necessary to obtain an extract with an even higher content of carotenoids, in addition to testing the hypothesis of antagonistic interaction between these classes of natural products. Thus, an optimized extract with a higher content of carotenoid, obtained from cashew apple bagasse, could be developed, focusing on the adjuvant treatment of intestinal inflammatory diseases.

## Figures and Tables

**Table 1:** Chemical characterization of the aqueous extract (CAE) obtained from cashew apple bagasse.

Chemical Constituents	CAE ^1^	
	mg/100 g	Relative Amount (%)
Anacardic acids		
C15:3	94.2 ± 0.6	-
C15:2	108.4 ± 0.1	-
C15:1	214.8 ± 0.2	-
Phenolic compound (total) ^2^	37.49 ± 0.64	-
Carotenoids		
Total ^3^	96.28 ± 0.15	
Non-provitamin A ^4^	-	76.9
Provitamin A ^4^	-	22.4

^1^ According to [24,25,26]. ^2^ Results expressed as mg gallic acid equivalent/100 g CAE [26]. ^3^ Results expressed as mg total carotenoids. ^4^ Results expressed based in the identified carotenoids [24].

**Table 2:** Estimated amount of the identified chemical constituent in CAE considering the administrated doses.

Chemical Constituents	CAE ^1^	
	100 mg/kg	500 mg/kg
Anacardic acids		
C15:3	1.9	9.4
C15:2	2.2	11
C15:1	4.3	21.5
Phenolic compound (total) ^2^	0.75	3.75
Carotenoids		
Total ^3^	1.93	9.63
Non-provitamin A ^4^	1.48	7.40
Provitamin A ^4^	0.43	2.16

^1^ According to [24,25,26]. ^2^ Results expressed as mg gallic acid equivalent/100 g CAE [26]. ^3^ Results expressed as mg total carotenoids. ^4^ Results expressed based in the identified carotenoids [24].

**Table 3:** Body weight evolution of female C57 BL/6J mice before DSS challenge.

Experimental Week	Parameters	Groups					
		G1	G2	G3-A	G4-A	G3-B	G4-B
Basal	Weight	19.90 ± 0.43	20.12 ± 0.25	21.08 ± 0.43	20.10 ± 0.56	20.21 ± 0.36	20.24 ± 0.41
1st	Weight	20.58 ± 0.52	20.74 ± 0.25	21.41 ± 0.34	20.87 ± 0.55	20.39 ± 0.34	20.91 ± 0.4
	∆	3.4 ± 1.8 ^a^	3.1 ± 2.5 ^a^	1.7 ± 3.4 ^a^	0.9 ± 1.3 ^a^	4.0 ± 4.8 ^a^	3.3 ± 1.8 ^a^
2nd	Weight	20.69 ± 0.43	20.54 ± 0.22	20.54 ± 0.22	20.52 ± 0.54	19.98 ± 0.44	20.84 ± 0.46
	∆	4.0 ± 1.2 ^a^	2.1 ± 2.2 ^a^	1.2 ± 2.4 ^a^	−1.1 ± 1.7 ^b,c^	2.1 ± 2.3 ^a,c^	2.9 ± 3.3 ^a^
3rd	Weight	21.23 ± 0.51	21.46 ± 0.19	21.60 ± 0.30	21.10 ± 0.42	20.47 ± 0.41	21.39 ± 0.28
	∆	6.6 ± 1.5 ^a^	6.7 ± 2.7 ^a^	2.6 ± 3.4 ^a,c^	1.4 ± 4.4 ^b,c^	5.2 ± 3.6 ^a,c^	5.8 ± 3.8 ^a^
4th	Weight	21.10 ± 0.38	21.42 ± 0.2	21.29 ± 0.41	21.11 ± 0.34	20.51 ± 0.42	21.42 ± 0.20
	∆	6.0 ± 1.5 ^a^	6.5 ± 2.5 ^a^	1.1 ± 3.9 ^b^	1.5 ± 2.9 ^b,c^	5.2 ± 3.6 ^a,c^	6.0 ± 3.9 ^a^

Results expressed as mean ± standard deviation. Animals: female C57BL/6J mice (8 weeks). Parameters: weight = body weight (g) on the basal, 1st, 2nd, 3rd, or 4th experimental week; ∆ = [(body weight (g) on the 1st, 2nd, 3rd, or 4th experimental week—basal body weight (g)/(basal body weight (g)) × 100]. Groups: G1—satellite group; G2—DSS-challenge group; G3-A and G4-A—37-day dose-repeated CAE treatment at 100 and 500 mg/Kg, respectively; G3-B and G4-B—4-day dose-repeated CAE treatment at 100 and 500 mg/Kg, respectively. Statistical analysis by two-way ANOVA followed by Bonferroni’s test (different letters indicate a significant difference in the same row, *p* ≤ 0.05).

## Data Availability

The dataset analyzed during the current study is available from the corresponding author Ana Lucia T. G. Ruiz on reasonable request.

## Supplementary Materials

The following supporting information can be downloaded at: https://www.mdpi.com/article/10.3390/foods12173318/s1, Figure S1: Effect of CAE on colon length in the DSS-induced colitis model in C57BL/6J mice; Figure S2: Representative photomicrographs of the histopathological analysis of the mouse colon in the DSS-induced acute colitis model; Table S1: Estimated average daily water consumption by C57BL/6J mice during the DSS challenge.
