# Supplementary material for: Evaluation of a Standardized Extract Obtained from Cashew Apple (Anacardium occidentale L.) Bagasse in DSS-Induced Mouse Colitis
Source: Foods. 2023 Sep 4;12(17):3318. doi: 10.3390/foods12173318 (PMC10486448; doi:10.3390/foods12173318)
Supplement: Supplementary file 1 [file foods-12-03318-s001.zip › foods-2550751-supplementary.pdf]

foods

Article – Supplementary Material

Evaluation of a standardized extract obtained from cashew apple (*Anacardium occidentale* L.) bagasse in DSS-induced mouse colitis

Gisele Goulart da Silva [1], Lucia Elaine de Oliveira Braga [1], Ellen Cristina Souza de Oliveira [2], João Ernesto de Carvalho [3], Josy Goldoni Lazarini [4,5], Pedro Luiz Rosalen [1,6], Ana Paula Dionísio [7], Ana Lucia Tasca Gois Ruiz [3,*]

[1] Piracicaba Dental School, Graduate Program in Dentistry, University of Campinas, UNICAMP, 13414-903, Piracicaba, SP, Brazil; giselegoulart87@gmail.com; naine17@hotmail.com; rosalen@fop.unicamp.br
[2] Institute of Biology, Cellular and Structural Biology Graduate Program, University of Campinas, UNICAMP, 13083-865, Campinas, SP, Brazil.; ellen_oliveira87@yahoo.com.br
[3] Faculty of Pharmaceutical Sciences, University of Campinas, UNICAMP, 13081-970 Campinas, SP, Brazil; carvalho@fcf.unicamp.br; ana.ruiz@ fcf.unicamp.br
[4] Piracicaba Dental School, Department of Physiological Sciences, University of Campinas, UNICAMP, 13414-903, Piracicaba, SP, Brazil; josy662@hotmail.com
[5] Faculty of Medicine, Universidade Anhembi Morumbi, 13425-380, Piracicaba, SP, Brazil; josy662@hotmail.com
[6] Biological Sciences Graduate Program, Federal University of Alfenas, UNIFAL-MG, 37130-001, Alfenas, MG, Brazil; rosalen@fop.unicamp.br
[7] Embrapa Agroindústria Tropical, 60511-110, Fortaleza, CE, Brazil; ana.dionisio@embrapa.br
* Correspondence: ana.ruiz@fcf.unicamp.br

Abstract: Inflammatory bowel diseases (IBD) include Crohn's disease and ulcerative colitis. Several studies relate eating habits to different aspects of IBD, such as progression and worsening of the clinical condition. Therefore, many natural products (NPs) such as polyphenols and carotenoids have been identified as promising agents in supporting IBD. An interesting source for obtaining bioactive NPs are the by-products of the food industry. The present study evaluated the potential beneficial effect of a standardized extract (CAE) obtained from cashew apple bagasse in the dextran sulfate sodium (DSS)-induced ulcerative colitis model in mice. This was the first time that CAE had been evaluated in this experimental model. Chemical evaluation of CAE identified carotenoids (96.28 ± 0.15 mg/100 g), phenolic compounds (37.49 ± 0.64 mg/100 g) and a mixture of anacardic acids (C15:3 = 94.2 ± 0.6 mg/100 g; C15:2 = 108.4 ± 0.1 mg/100 g; C15:1 = 214.8 ± 0.2 mg/100 g). Administration of CAE (500 mg/kg, 4 days, p.o.) after DSS challenge was more effective in delaying disease progression compared with prior treatment (500 mg/kg, 30 days, p.o.), according to the disease activity index. However, no treatment strategy with CAE was able to prevent or inhibit disease progression, since all parameters evaluated (macroscopic, biochemical and histopathological) in CAE-treated animals were similar to those observed in DSS-challenged animals. Despite the high dose (500 mg/kg), the standardized extract (CAE) did not result in an effective concentration of carotenoids. Furthermore, as some anacardic acids have been reported as histone acetyltransferases inhibitors, there could be a possible antagonistic relationship between carotenoids and anacardic acids. Complementary research will be necessary to test the hypothesis of antagonism. Thus, an optimized extract, with an even higher concentration of carotenoids, obtained from cashew apple bagasse, can be developed as a possible adjuvant food supplement for inflammatory bowel diseases.

Keywords: *Anacardium occidentale* L.; food by-products; carotenoids, anacardic acids; colitis, inflammatory bowel diseases.

Table S1. Estimated average daily water consumption by C57BL/6J mice during the DSS challenge.

Group	Water volume (ml)/animal/day						
	1	2	3	4	5	6	7
Satellite (G1)	6.4	4.6	5.4	7.6	5.8	7.2	5.4
DSS (G2 – G4)	4.3	4.0	4.4	3.3	2.9	6.0	1.6

Results expressed as an estimated volume = [(volume at the experimental day - volume at the previous experimental day)/number of animals per cage]. Animals: female C57 BL/6J mice (8 weeks). Group G1: 5 animals accommodated in one cage; groups G2 - G4: n = 7 animals/group accommodated 5 animals per cage, totaling 7 cages. Challenge: Satellite = drinking water ad libitum (group G1); DSS = dextran sulfate sodium solution (3%) in drinking water ad libitum from 30th to 37th experimental day (groups G2 - G4). Treatment: G3-A and G4-A = 37-day dose-repeated CAE treatment at 100 and 500 mg/kg; G3-B and G4-B = 4-day dose-repeated CAE treatment at 100 and 500 mg/kg.

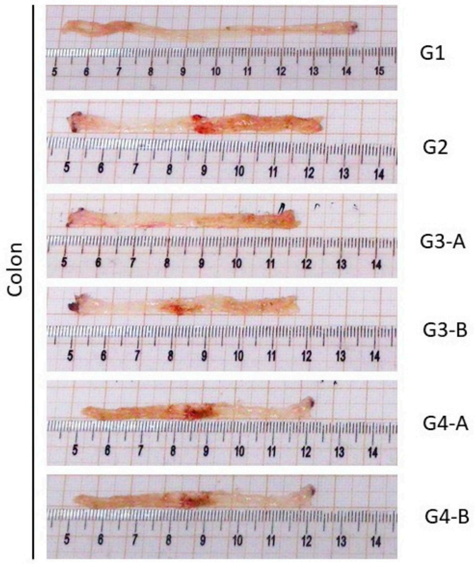

Figure 1. Effect of CAE on colon length in the DSS-induced colitis model in C57BL/6J mouse.

Representative images of the macroscopic analysis of the female C57BL/6J mice colon after DSS challenge. Challenge: Satellite = drinking water ad libitum (group G1); DSS = dextran sulphate sodium solution (3%) in drinking water ad libitum from 30th to 37th experimental day (groups G2 - G4). Treatment: G3-A and G4-A = 37-day dose-repeated CAE treatment at 100 and 500 mg/kg; G3-B and G4-B = 4-day dose-repeated CAE treatment at 100 and 500 mg/kg.

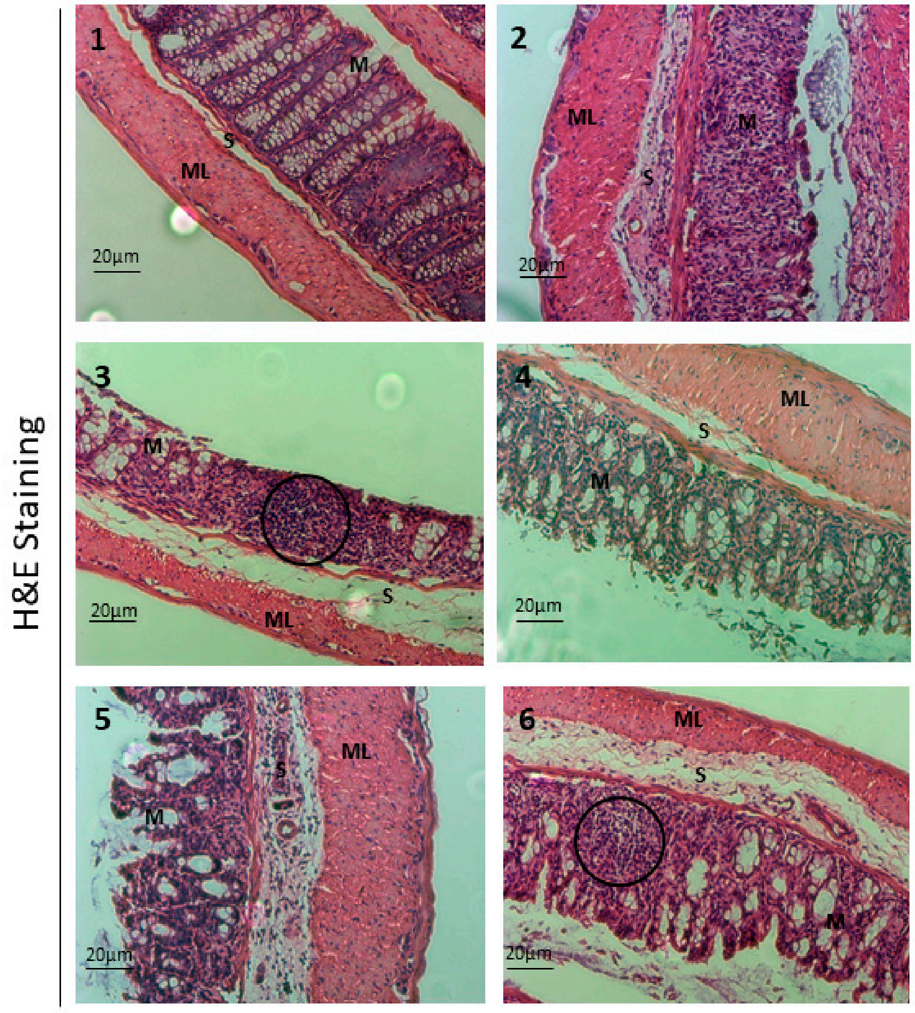

Figure S2. Representative photomicrographs of the histopathological analysis of the mouse colon in the DSS-induced acute colitis model. (**1**) Satellite (group G1); (**2**) DSS challenge (group G2); (**3**) and (**4**) 37-day dose-repeated CAE treatment at 100 and 500 mg/Kg (groups G3-A and G4-A), respectively, plus DSS challenge; (**5**) and (**6**) 4-day dose-repeated CAE treatment at 100 and 500 mg/Kg (groups G3-B and G4-B), respectively, plus DSS challenge. Histological findings: Mucosa (M), submucosa (S), Muscle (ML), Circles indicate ulcerations.
